# Nanocrystals Incorporated with Mordenite Zeolite Composites with Enhanced Upconversion Emission for Cu^2+^ Detection

**DOI:** 10.3390/ma17040854

**Published:** 2024-02-11

**Authors:** Peixuan Lin, Song Ye, Ling Pan, Ruihao Huang, Haoran Zhang, Deping Wang

**Affiliations:** School of Materials Science and Engineering, Tongji University, Shanghai 201804, China; 2133751@tongji.edu.cn (P.L.); 2130648@tongji.edu.cn (L.P.); 2232898@tongji.edu.cn (R.H.); 2232891@tongji.edu.cn (H.Z.); wdpshk@tongji.edu.cn (D.W.)

**Keywords:** upconversion nanocrystals, MOR zeolites, luminescence properties, fluorescent probes

## Abstract

In this research, upconversion nanocrystals incorporated with MOR zeolite composites were synthesized using the desilicated MOR zeolite as a host for the in situ growth of NaREF_4_ (RE = Y, Gd) Yb/Er nanocrystals. The structure and morphology of the composites were studied with XRD, XPS, and TEM measurements, and the spectral studies indicated that the subsequent thermal treatment can effectively improve the upconversion emission intensity of Er^3+^. By using the NaYF_4_:Yb/Er@DSi1.0MOR-HT composite that holds the strongest upconversion emission, a probe of UCNC@DSiMOR/BPEI was constructed with the modification of branched poly ethylenimine for the detection of Cu^2+^. It was indicated that the integrated emission intensity of Er^3+^ shows a linear dependence with the logarithm value of the Cu^2+^ concentration ranging from 0.1 to 10 μM. This study offered a feasible method for the construction of UCNC@zeolite composites with enhanced upconversion emission, which may have a potential application as fluorescent probes for the detection of various metal ions by adjusting the doping luminescent center.

## 1. Introduction

Among various photoluminescent materials, such as quantum dots, inorganic phosphors, metal–organic frameworks, and organic dyes, the lanthanide-doped upconversion nanocrystals (UCNCs) that can convert near-infrared light into short-wavelength light in the visible range have attracted great research interest. The unique properties of large anti-Stokes shifts, narrow emission peaks, and the superior photo stability of the lanthanide-doped UCNCs enable them to be applied in sensors, detection, displays, and lasers [1,2,3].

Multiple emissions of UCNCs can be readily realized to satisfy the application requirements by selecting the rare earth (RE) activator, for example, Tm^3+^ for blue emission and Er^3+^ or Ho^3+^ for red and green emissions [4,5]. In addition, the structure and composition of the host nanocrystal are key factors to generate efficient upconversion emissions. Previous studies strongly suggested that NaYF_4_ and NaGdF_4_ are desirable hosts due to their low-phonon energy that can effectively suppress the non-radiative relaxation, high solubility for RE ions, and high thermal stability [6,7]. Another common problem encountered by the small UCNCs is the strong surface luminescence quenching due to a high surface to volume ratio [8,9,10]. The coating of the inert shell on the active core UCNCs is a generally used method to spatially separate the activators and the surface quenching centers and thus greatly improve the upconversion emission intensity [11,12]. It was reported that the lanthanide-doped NaGdF_4_ is wrapped with a CaF_2_ shell on its surface, and the heterogeneous structure of the CaF_2_ shell greatly enhances its upconversion emission [13]; the growing of an inert NaGdF4 shell can effectively improve the upconversion emission intensity of NaYF4:Yb,Er@NaGdF4:Yb,Nd core–shell UCNCs under both 808 and 980 nm excitations [14]. Furthermore, the construction of UCNC-based nano–micro composites is an alternative strategy to obtain desirable upconversion emissions and even realize new functions. For example, the upconversion nanocrystals were combined with metal–organic frameworks (MOFs) to improve their luminescence performance for theranostic applications [15]; the flexible polystyrene (PS) sphere array @UCNC composite was fabricated via self-assembly to promote the upconversion process for the sensing of acetic acid gas [16]. Besides MOFs and organics, the UCNCs can also be combined with various inorganics to construct functional composites for various potential applications.

Zeolites are crystalline aluminosilicates with more than 200 topology structures, which have been widely applied in the fields of catalysis, adsorption, and drug delivery. In recent years, many of the zeolites have received great research interest for the synthesis of functional composites because of their regular porous framework, which is capable of incorporating various nanoparticles [17,18,19]. It was reported that zeolites can be used as a matrix for various nano-sized metal and oxides to construct nanoparticle@zeolite composites, which not only improved the properties of individual nanoparticles but also endowed the composites with new functions [20]. For example, Pd nanoparticles were successfully encapsulated in MCM-41, selective thermal treatment during synthesis via the microwave method allowed Pd nanoparticles to enter into zeolite pores without aggregating on the outside, and, meanwhile, the crystal structure of the zeolite was not altered after microwave treatment, which provided a novel path for the design and development of composite materials and excellent size selectivity in catalytic reactions [21]. An LTA zeolite doped with titanium dioxide was prepared via the hydrothermal method and used as electrochemical catalyst for detecting H_2_O_2_ [22]. Generally, the pore size of pristine zeolites is in the molecular scale of 0.3–1.5 nm, which may limit their application as carriers for the incorporation of nanoparticles [23]. Thereafter, some post-treatments like desilication and dealumination have been applied to enlarge the pore size and create more mesopores to benefit the loading of various nanoparticles [24,25,26]. For example, the dealuminated Zeolite 13X was used to load CeO_2_ nanoparticles to prepare composites for the effective degradation of caffeine [27] and the desilicated FAU-Y and ZSM-5 zeolites were used as target zeolites for the in situ growth of UCNCs for gallic acid detection or drug delivery monitoring [14,28]. A benefit for the flexible control of the upconversion emission spectral profile is that by selecting the luminescence center, like Tm^3+^, Ho^3+^, and Er^3+^, it is expected that the UCNC-loaded zeolites can be developed as sensitive probes for the detection of various chemicals in aqueous solutions.

The monitoring of the Cu^2+^ level in water is an important environmental issue, and fluorescence spectroscopy can be used to detect metal ions with a high sensitivity, low cost, and simplicity. So far, fluorescent probes have been developed for the selective detection of Cu^2+^, like the rhodamine derivative of poly ethylene glycol (PEG)- neridronate, rhodamine B hydrazide (RBH), and ethylene imine polymer (PEI) modified UCNCs [29,30,31]. In this research, UCNCs incorporated with MOR zeolite composites of UCNC@DSiMOR were synthesized using the desilicated MOR zeolite as host for the in situ growth of NaREF_4_ (RE = Y, Gd) Yb^3+^/Er^3+^ nanocrystals, which showed improved upconversion emissions after further thermal treatment. Using branched poly ethylenimine (BPEI) combined with NaYF_4_:Yb/Er@DSi1.0MOR-HT as a fluorescent probe, the level of Cu^2+^ in water can be effectively and accurately detected.

## 2. Materials and Methods

### 2.1. Materials

The sodium form mordenite zeolite was supplied by Thermo Scientific Chemical Co., Ltd, Shanghai, China. YCl_3_·6H_2_O (99.99%), YbCl_3_·6H_2_O (99.99%), ErCl_3_·6H_2_O (99.99%), GdCl_3_·6H_2_O (99.99%), NH_4_F (99.99%), oleic acid (OA, 90%), sodium oleate (NaOA, >97%), 1-octadecene (ODE, 90%), sodium hydroxide (NaOH, 97%), CuCl_2_·2H_2_O (AR), Na_2_SO_4_ (AR), ethanol (AR), methanol (99.9%), and cyclohexane (AR) were purchased from Aladdin Chemical Co., Ltd. FeCl_3_·6H_2_O (AR), MnCl_2_·4H_2_O (AR), MgCl_2_·6H_2_O (AR), CaCl_2_ (AR), KCl (AR), NaCl (AR), NaF (AR), Na_2_CO_3_ (AR), and hydrochloric acid (HCl, AR) were purchased from Sinopharm Chemical Reagent Co., Ltd., Shanghai, China. The branched poly ethylenimine (BPEI, M.W. 25,000, 30% in water) was purchased from Tansolo Co., Ltd., Shanghai, China. All chemical reagents were used without further purification.

### 2.2. Desilication of Mordenite Zeolite

The mordenite zeolite was modified through desilication upon alkaline treatment in NaOH solution to increase the porosity. Each 1 g of the parent zeolite was stirred in 30 mL of NaOH aqueous solution with different concentrations of 1.0 and 2.0 mol/L at 85 °C for 2 h. After that, the obtained suspensions were cooled down to room temperature, filtered, and washed with deionized water. The final products were collected via centrifugation before drying at 60 °C for 24 h. According to the NaOH aqueous solution concentration, the alkali-treated zeolites were labeled as DSi*x*MOR, where *x* = 1.0, 2.0, respectively. The purchased sodium form mordenite zeolite was labeled MOR.

### 2.3. Synthesis of NaREF_4_:Yb/Er (RE = Y, Gd) @DSixMOR Composites

The UCNC@DSi*x*MOR composite was prepared via the impregnation of DSi*x*MOR in the synthesis of NaYF_4_:Yb/Er nanocrystals with the coprecipitation method, for the growth of NaYF_4_:Yb/Er in the cages of DSi*x*MOR. In the first step, 1 mmol of RE chlorides was mixed with 6 mL OA and 15 mL ODE in a three-necked flask. The RE chlorides included 0.18 mmol YbCl_3_·6H_2_O, 0.02 mmol ErCl_3_·6H_2_O, and 0.8 mmol YCl_3_·6H_2_O. After that, the solution was mixed for 30 min in 160 °C under an argon atmosphere to dissolve the RE chlorides. After cooling down to 40 °C, 0.25 g of DSi*x*MOR was added into the flask and stirred for 90 min. Then, 10 mL methanol solution of NaOA (0.7611 g) and 10 mL methanol solution of NH4F (0.1482 g) were added into this solution respectively. The solution was slowly heated up to 65 °C and all of the methanol was removed. Next, the solution was heated for 30 min at 280 °C under an argon atmosphere. After the solution was cooled down to room temperature, the composites were collected via centrifugation and washed with ethanol three times before being dried at 60 °C for 24 h. Finally, the obtained composites were heat-treated at 400 °C for 150 min to obtain an improved upconversion emission from Er^3+^. The resulting composites were named as NaYF_4_:Yb/Er@DSi*x*MOR and NaYF_4_:Yb/Er@DSi*x*MOR-HT, respectively. 

NaGdF_4_:Yb/Er@DSi*x*MOR and NaGdF_4_:Yb/Er@DSi*x*MOR-HT were synthesized using GdCl_3_·6H_2_O instead of YCl_3_·6H_2_O at the initial synthesis step; the other synthesis conditions were the same.

### 2.4. Detection of Cu^2+^

#### 2.4.1. Surface Modification of NaYF_4_:Yb/Er@DSi1.0MOR-HT Composite

For the preparation of the luminescence probe, the NaYF_4_:Yb/Er@DSi1.0MOR-HT was modified with BPEI. Firstly, the NaYF_4_:Yb/Er@DSi1.0MOR-HT was added into 0.05 M HCl solution with stirring for 20 h to remove the oleate ligand from the surface and then washed with water 3 times. After centrifugation, the solids were added into water followed by the addition of 0.4 mM BPEI and stirring for 20 h at 25 °C to modified BPEI on NaYF_4_:Yb/Er@DSi1.0MOR-HT. Finally, NaYF_4_:Yb/Er@DSi1.0MOR-HT/BPEI composites were collected via centrifugation, washed with water, and dried at 60 °C for 24 h. The resulting samples were named UCNC@DSiMOR/BPEI.

#### 2.4.2. Detection of Cu^2+^ by Using the NaYF_4_:Yb/Er@DSi1.0MOR-HT/BPEI Composite

In terms of the detection of Cu^2+^, NaYF_4_: Yb/Er @DSi1.0MOR-HT/BPEI composites were added into different concentrations (0.1 to 10 μM) of CuCl_2_·2H_2_O solutions. Then, the upconversion spectra of luminescence probes were measured under 980 nm excitation. Additionally, to indicate the principle of detection, we added 0.4 mM BPEI into 1 mM CuCl_2_·2H_2_O solution and measured the UV-Vis NIR absorption spectra.

#### 2.4.3. Anti-Interference Test

To validate the sensitivity and selectivity for the probing of Cu^2+^ in aqueous solutions, various potential interference substances, including Fe^3+^, Mn^2+^, Mg^2+^, Ca^2+^, K^+^, Cl^−^, F^−^, CO_3_^2−^, and SO_4_^2−^, were chosen for an anti-interference test of UCNC/BPEI. For the fluorometric assay, all the other conditions were kept the same except that the concentrations of metal ions and the anion solution were settled to 100 μM. The probe without adding any substances was set as a blank control.

### 2.5. Characterization

X-ray diffraction (XRD) analyses were recorded on a Rigaku Smartlab9 diffractometer with Cu-Kα radiation (λ = 1.5406 Å, 40 KV/150 mA). The N_2_ adsorption–desorption isotherm of the zeolite samples was measured via a Micromeritics ASAP2020, Atlanta, GA, USA, at 77 K. And the total surface area was calculated using the Brunauer–Emmett–Teller (BET) method, while the t-plot method was used to determine the surface area of micropores and mesopores. The pore size distribution was derived from the Barrett–Joyner–Halenda (BJH) model. X-ray photoelectron spectroscopy (XPS) characterization was carried out on a Thermo ESCALAB 250XI using Al Kα X (ℎν = 1486.6 eV, 650 μm of beam spot) as the incident radiation source, and the electron flood gun was used to minimize surface charging. The ion content in the final alkaline treatment solution was determined via inductively coupled plasma optical emission spectroscopy (ICP-OES), (ICP-8300, PerkinElmer, America). Transmission electron microscope (TEM) analyses were carried out on a JEM-2100F TEM instrument. The upconversion spectra were measured using a set of well-aligned instruments (Zolix Instruments Co. Ltd.), and the 980 nm laser was used as excitation source. The UV-Vis NIR absorption spectra were recorded using a fluorescence spectrophotometer (U-4100).

## 3. Results and Discussion

### 3.1. Structure and Morphology

**Figure 1:**
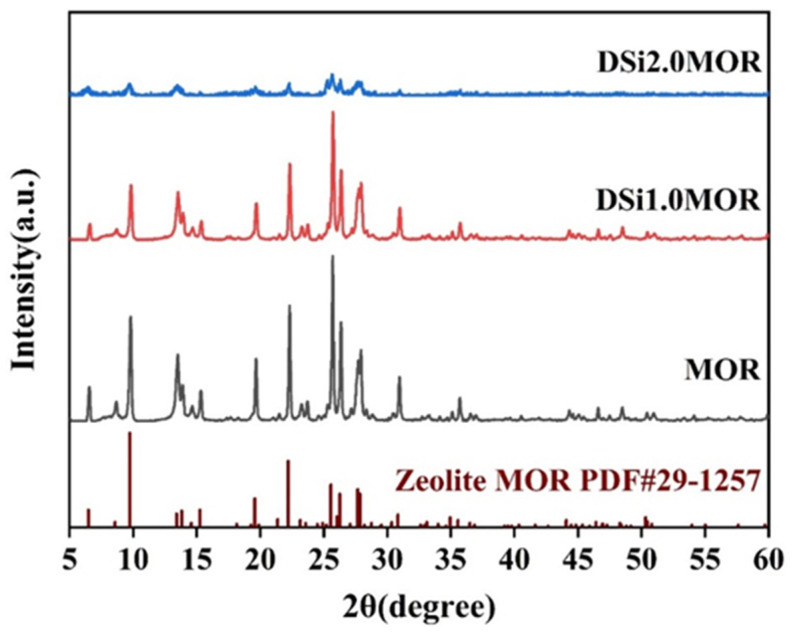
XRD patterns of MOR zeolite and DSixMOR (x = 1.0, 2.0) zeolite.

The influence of desilication on the structure of the parent MOR zeolite was firstly studied with XRD measurement and is shown in Figure 1. The XRD patterns of DSi1.0MOR and DSi2.0MOR are in accordance with that of the parent zeolite; it is noticable that the diffraction peak intensity of DSi1.0MOR is very strong, while that of DSi2.0MOR is extremely weak, which indicates that the alkali treatment at a concentration of 2.0 M had already destroyed the zeolite structure. Therefore, DSi1.0MOR was chosen as the target zeolite for the subsequent UCNC growth. DSi1.0MOR ensures a well-defined structure for the construction of composites incorporating UCNCs, while mesopores provide space for the growth of UCNCs without structural constraints. It can be seen from Table 1 that DSi1.0MOR exhibits a decreased specific surface area, but its mesopore surface area, mesopore volume, and average mesopore size are increased, which suggested the successful creation of enlarged mesopores upon alkali treatment [32,33]. The removal of Si atoms from the framework led to the creation of voids and larger cavities within the zeolite structure. Compared with the parent MOR zeolite, DSi1.0MOR can offer improved accessibility for larger molecules. This is crucial for applications like adsorption and detection, where bulky reactants or products need efficient diffusion within the probe materials.

**Figure 2:**
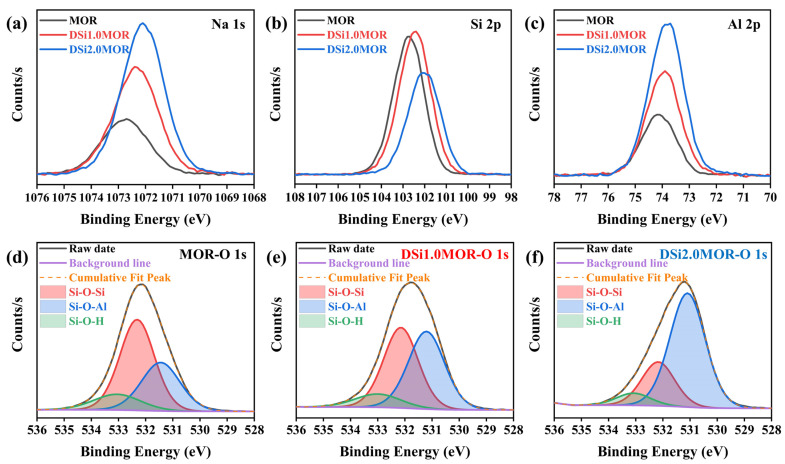
The high-resolution XPS spectra for Na 1s (**a**), Si 2p (**b**), Al 2p (**c**), and O 1s (**d**–**f**) in MOR zeolite and DSi*x*MOR (*x* = 1.0, 2.0) zeolite.

**Figure 3:**
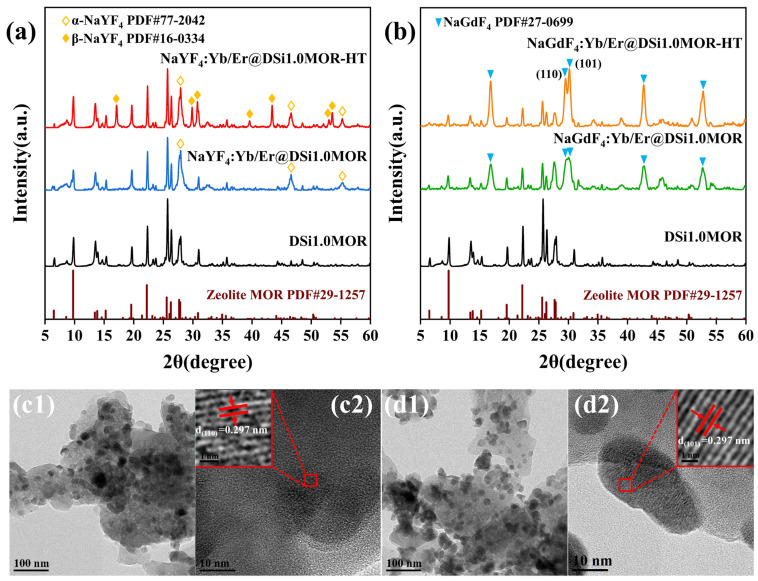
XRD patterns of DSi1.0MOR, NaREF_4_:Yb/Er@DSi1.0MOR, and NaREF_4_:Yb/Er@DSi1.0MOR-HT (RE = Y, Gd) (**a**,**b**); TEM images of NaREF_4_:Yb/Er@DSi1.0MOR-HT (RE = Y, Gd) (**c**,**d**).

For further understanding the effect of alkali treatment on the elemental composition and chemical state of the parent MOR zeolite, the XPS measurement was carried out on MOR, DSi1.0MOR, and DSi2.0MOR, respectively. Figure 2a–f exhibit the high-resolution XPS spectra of Na 1s, Si 2p Al 2p, and O 1s, according to which the atomic percentages of each element were calculated, which are listed in Table 2. It can be seen that the atomic percentage of Si decreases in the alkali-treated zeolites and the corresponding Si/Al ratio obviously decreased in DSi1.0MOR and DSi2.0MOR, suggesting the successful removal of Si from the parent MOR zeolite. This is also consistent with the monotonously increased Si concentration in the alkaline treatment solution after a reaction measured using ICP (Table S1). Moreover, it is noticed from the high-resolution XPS spectra that the photoelectron peaks of Na 1s, Si 2p Al 2p, and O 1s all showed negative shifts in DSi1.0MOR and DSi2.0MOR compared with in the parent MOR zeolite; the binding energies of Na 1s, Si 2p Al 2p, and O 1s all shift in the same direction with changes in the Si/ Al ratio. This binding energy shift can be explained in terms of a charge transfer in the zeolite lattice. Zeolite desilication causes the removal of Si atoms and the breaking of Si-O-Si bonds and increases the Si-O-Al bond percentage of the zeolite framework. As shown in Figure 3d–f, the high-resolution XPS spectra of O 1s can be deconvoluted into three components that can be attributed to Si-O-Si, Si-O-Al, and Si-O-H, respectively. As the concentration of the NaOH solution increases, the proportion of Si-O-Si bonds decreases and the proportion of Si-O-Al bonds increases, accompanied by a decrease in Si-O-H bonds, indicating that the removal of Si atoms lead to an increased Si-O-Al bond percentage [34,35]. It is estimated that the Si-O-Al percentages in MOR, DSi1.0MOR, and DSi2.0MOR are 32.6%, 45.3%, and 68.4%, respectively. In this case, the negative binding energy shifts of Na 1s, Si 2p, Al 2p, and O 1s of the desilicated MOR zeolites can be attributed to the increase in negative charge due to the increase in Si-O-Al bonds, and the lower the Si/Al ratio, the more negative the binding energy shift [36,37].

After the in situ growth of UCNCs in the DSi1.0MOR zeolite and the subsequent thermal treatment, the structures of the compounds were firstly characterized with XRD measurements. As shown in Figure 3a, the XRD patterns of NaYF_4_:Yb/Er@DSi1.0MOR and NaYF_4_:Yb/Er@DSi1.0MOR-HT both maintain the typical diffraction peaks belong to the MOR zeolite, and meanwhile the diffraction peaks only assigned to α-NaYF_4_ and assigned to both α-NaYF_4_ and β-NaYF_4_ nanocrystals can be observed before and after the thermal treatment, respectively, due to the heat treatment-induced phase transition [38]. The XRD patterns for NaGdF_4_:Yb/Er@DSi1.0MOR and NaGdF_4_:Yb/Er@DSi1.0MOR-HT composites show similar characteristics, which both keep the diffraction peaks belong to the MOR zeolite together with additional diffraction peaks assigned to NaGdF_4_. It is noticed that the (110) and (101) plane diffractions of NaGdF_4_ can be clearly discriminated after thermal treatment, indicating an increased crystal size. The TEM images of NaYF_4_: Yb/Er @DSi1.0MOR-HT and NaGdF_4_: Yb/Er @DSi1.0MOR-HT in Figure 3c,d both show some black dots on DSi1.0MOR substrates, and no free nanoparticles can be observed. The locally magnified high-resolution TEM images reveal the fine crystalline structure of the black dots, as shown in Figure 3(c2,d2); the lattice spacing of 0.297 nm well corresponds to the (110) plane of NaYF_4_, and the lattice space of 0.297 nm is in good accordance with the (101) plane of NaGdF_4_.

**Figure 4:**
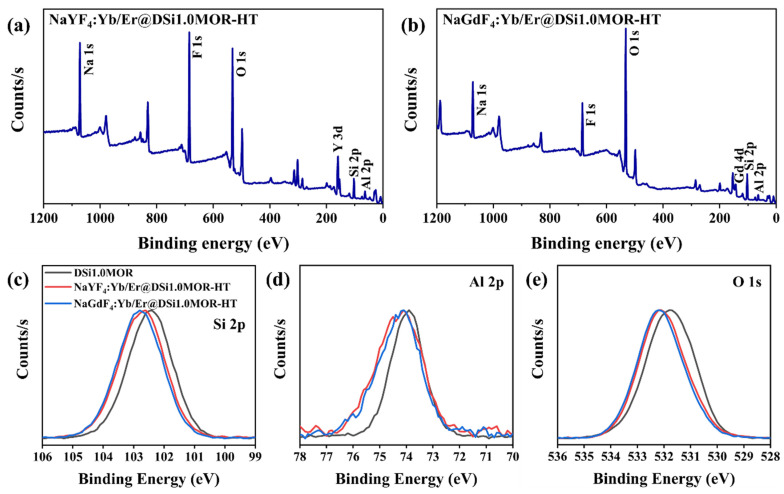
The XPS survey spectra for NaYF_4_:Yb/Er@DSi1.0MOR-HT (**a**) and NaGdF_4_:Yb/Er@DSi1.0MOR-HT (**b**); high-resolution XPS spectra for Si 2p (**c**), Al 2p (**d**), and O 1s (**e**) in DSi1.0MOR, NaYF_4_:Yb/Er@DSi1.0MOR-HT, and NaGdF_4_:Yb/Er@DSi1.0MOR-HT.

Moreover, the XPS measurement was carried out and the survey spectra of NaREF_4_:Yb/Er@DSi1.0MOR (RE = Y, Gd) are shown in Figure 4a,b, from which the Na 1s, Si 2p, Al 2p, and O 1s photoelectron peaks belonging to DSi1.0MOR and the F 1s, Y 3d, and Gd 4d photoelectron peaks belonging to NaREF_4_ can be observed, respectively. The high-resolution XPS spectra of Si 2p, Al 2p, and O 1s in Figure 4c–e show that the photoelectron peaks of the elements that constitute the host zeolite all positively shift in NaREF_4_:Yb/Er@DSi1.0MOR-HT compared with those in DSi1.0MOR. The increased banding energies of Si 2p, Al 2p, and O 1s in NaREF_4_:Yb/Er@DSi1.0MOR (RE = Y, Gd) composites imply that the constitution ions of DSi1.0MOR have undergone a charge transfer with the NaREF_4_:Yb/Er nanocrystal and therefore the existing chemical complex between them [39,40,41].

### 3.2. Upconversion Properties

**Figure 5:**
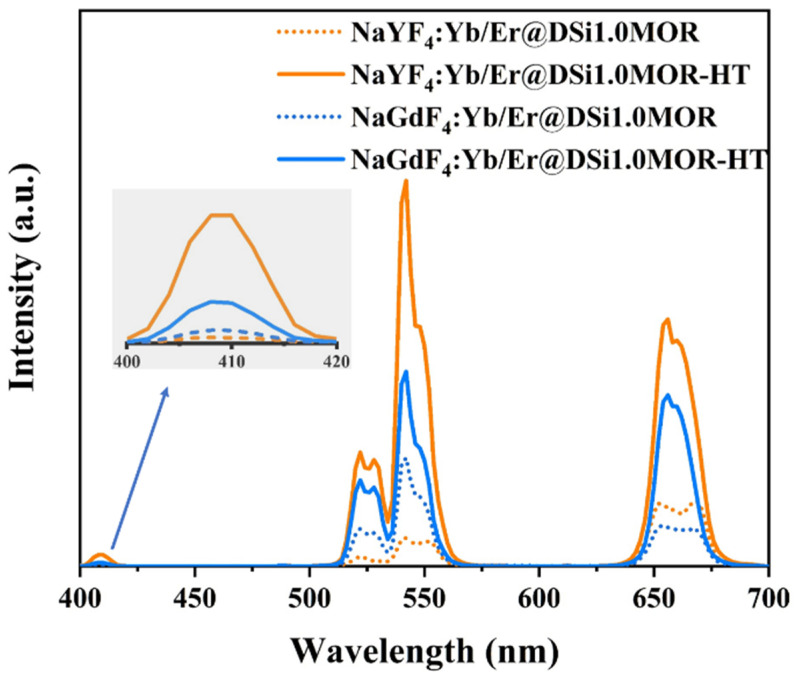
Upconversion spectra of NaREF_4_:Yb/Er@DSi1.0MOR and NaREF_4_:Yb/Er@DSi1.0MOR-HT (RE = Y, Gd).

Under the excitation of a 980 nm laser, the upconversion luminescence spectrum of NaREF_4_:Yb/Er@DSi1.0MOR exhibits a characteristic emission of Er^3+^ in the visible region, in which the emission peaks located at 521, 542, and 654 nm can be well attributed to the radiative transitions of Er^3+^: ^2^H_11/2_→^4^I_15/2_, ^4^S_3/2_→^4^I_15/2_, and ^4^F_9/2_→^4^I_15/2_, respectively, as shown in Figure 5 [42,43]. After heat treatment, the upconversion emission intensity of Er^3+^ is drastically enhanced; even Er^3+^: ^2^H_9/2_→^4^I_15/2_ radiative transition at 409 nm can be clearly observed in NaREF_4_:Yb/Er@DSi1.0MOR-HT. It is estimated from the emission spectra that the heat treatment-induced enhancements in emission intensity are 4.9 and 2.2 times for NaYF_4_:Yb/Er@DSi1.0MOR and NaGdF_4_:Yb/Er@DSi1.0MOR, respectively. According to previous studies, proper thermal treatment can promote the formation of tightly combined interfaces between the nanocrystals and the host zeolite, which can effectively modify the surface defects of the nanocrystals, and thus the improved upconversion emission can be obtained [28]. However, the increase in nanocrystal size after thermal treatment can also reduce the non-radiative relaxation probability of the surface RE ions and contributes to the strong upconversion emission [14,44]. As shown in Figure 5, the upconversion emission intensity of Er^3+^ is strongest in NaYF_4_:Yb/Er@DS1.0MOR-HT among these four considered samples.

### 3.3. Detection of Cu^2+^ via Upconversion Emission

**Figure 6:**
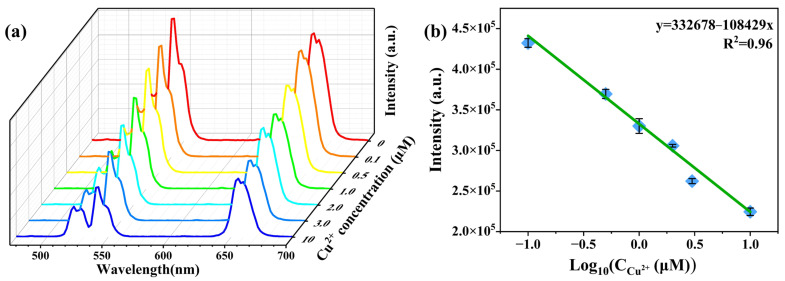
Upconversion integrated emission intensities of NaYF_4_:Yb/Er@DSi1.0MOR-HT/BPEI probe with increasing concentrations of Cu^2+^ under 980 nm excitation (**a**); the relationship between upconversion emission intensity and the concentrations of Cu^2+^ (**b**).

In the present research, we used the NaYF_4_:Yb/Er@DSi1.0MOR-HT composite that holds the strongest upconversion emission to construct an example probe of UCNC@DSiMOR/BPEI and to demonstrate the detection of Cu^2+^. The emission spectra of UCNC@DSiMOR/BPEI and the absorption spectra of the BPEI solution and BPEI-Cu^2+^ complex solution are shown in Figure S1. After BPEI was added to the solution of Cu^2+^, the amino groups of BPEI on the surface of UCNC@DSiMOR/BPEI coordinate with Cu^2+^, which shows an absorption band between 450 and 700 nm [45]. Upon excitation with a 980 nm laser, UCNC@DSiMOR/BPEI exhibits three emission peaks centered at 521, 542, and 654 nm, which can be attributed to Er^3+^: ^2^H_11/2_→^4^I_15/2_, ^4^S_3/2_→^4^I_15/2_, and ^4^F_9/2_→^4^I_15/2_ transitions, respectively. According to the spectra in Figure S1, the absorption spectrum of BPEI-Cu^2+^ complexes overlaps with the emission spectra of UCNC@DSiMOR. Therefore, the emission of UCNC@DSiMOR will be quenched in the presence of BPEI-Cu^2+^, especially for the red emission wavelength region. In order to obtain the relationship between the upconversion emission intensity of the UCNC@DSiMOR/BPEI probe and the Cu^2+^ level in water, the upconversion emission spectra of Er^3+^ were measured upon the addition of different concentrations of Cu^2+^, and the corresponding results are shown in Figure 6a. In order to reduce the errors caused by experimental and environmental factors, the spectral measurements of the samples for the detection of Cu were repeated three times using the same method, and the repeatability deviations are shown in Figure 6b. We can observe that the upconversion emission intensity monotonously decreases with increases in the Cu^2+^ concentration, and, meanwhile, the integrated emission intensity shows a linear dependence with the logarithm value of the Cu^2+^ concentration ranging from 0.1 to 10 μM. The limit of detection (LOD) is defined as 3 s/k, where s represents the standard deviation of the blank and k represents for the slope of the linear calibration equation. Here, the LOD of the UCNC@DSiMOR/BPEI probe was determined to be 1.507 μmol/L, which is comparable to or lower than some of the previously reported detection limits for Cu^2+^ (Table S2) [46,47,48,49,50,51,52].

**Figure 7:**
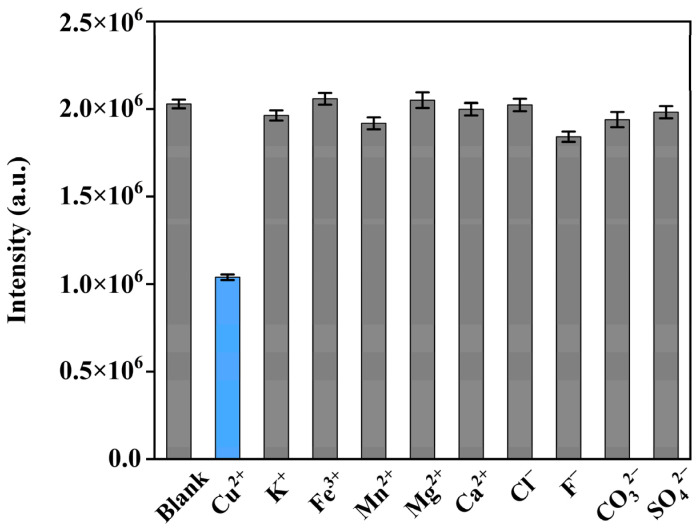
Selectivity of NaYF_4_:Yb/Er@DSi1.0MOR-HT/BPEI probe for Cu^2+^ over other representative metal ions and anions in aqueous solution.

Practical applications often contain a variety of ions and molecules that can interfere with Cu^2+^ detection. In order to validate the selectivity for the detection of Cu^2+^ using the UCNC@DSiMOR/BPEI as probe, various potential interference metal ions and ionic clusters, including K^+^, Ca^2+^, Mg^2+^, Mn^2+^, Fe^3+^, Cl^−^, F^−^, CO_3_^2−^, and SO_4_^2−^, were chosen for an anti-interference test, in which the concentrations of the interference ions’ aqueous solutions were set to 100 μM. The corresponding integrated upconversion emission intensities upon the adding of each interference solution are shown in Figure 7. To minimize errors due to experimental and environmental factors, the spectral measurements were repeated three times using the same method, and the repeatability deviations are also shown. It can be seen that compared with Cu^2+^, the presence of the considered interference ions and ionic clusters did not lead to an obvious decrease in the upconversion emission intensity of the probe. These results demonstrated the excellent sensitivity and selectivity of UCNC@DSiMOR/BPEI toward the detection of Cu^2+^.

## 4. Summary

In summary, the UNCN@MOR zeolite composites were successfully synthesized using an MOR zeolite desilicated with a 1.0 mol/L NaOH alkaline solution as the host for the in situ growth of NaREF_4_ (RE = Y, Gd) Yb/Er nanocrystals with the coprecipitation method, and the subsequent thermal treatment was conducted to effectively improve the upconversion emission intensity of Er^3+^. The NaYF_4_:Yb/Er@DSi1.0MOR-HT composite that holds the strongest upconversion emission was used to construct the UCNC@DSiMOR/BPEI probe for the selective detection of Cu^2+^ in water. The spectra results indicated that the integrated emission intensity of Er^3+^ holds a linear dependence with the logarithm value of the Cu^2+^ concentration ranging from 0.1 to 10 μM with an LOD of 1.507 μmol/L, while less emission quenching was exhibited in the presence of various potential interference metal ions and ionic clusters with a concentration of 100 μM. It is expected that the NaYF_4_:Yb/Er@DSi1.0MOR-HT composite can be a desirable probe for the detection of Cu^2+^ with good sensitivity and selectivity.

## Figures and Tables

**Table 1:** Surface area, pore volume, and average pore size for MOR and DSi1.0MOR zeolites.

Samples	Surface Area (m^2^/g)			Pore Volume (cm^3^/g)			Average Pore Size (nm)		
	Meso	Micro	BET	Meso	Micro	Total	Meso	Micro	BET
MOR	1	320	321	0.020	0.147	0.167	5.98	0.51	2.08
DSi1.0MOR	20	73	93	0.061	0.034	0.095	8.05	0.99	4.06

**Table 2:** XPS data of elemental composition of parent MOR zeolite, DSi1.0MOR, and DSi2.0MOR.

Sample	XPS Atomic (%)				Si/Al
	Si	Al	Na	O	
MOR	25.88	5.11	5.41	63.61	5.06
DSi1.0MOR	23.45	6.73	8.67	61.14	3.48
DSi2.0MOR	17.26	9.88	12.08	60.78	1.75

## Data Availability

The original contributions presented in the study are included in the article/supplementary material, further inquiries can be directed to the corresponding author/s.

## Supplementary Materials

The following supporting information can be downloaded at https://www.mdpi.com/article/10.3390/ma17040854/s1, Figure S1: Upconversion spectrum of NaYF_4_:Yb/Er@DSi1.0MOR-HT composite interacting with BPEI and absorption spectrum of BPEI and BPEI-Cu(II); Table S1: The ion concentrations in the alkaline treatment solution after reaction measured using ICP; Table S2: The limit of detection (LOD) of Cu^2+^.
