# Supplementary material for: Nanocrystals Incorporated with Mordenite Zeolite Composites with Enhanced Upconversion Emission for Cu2+ Detection
Source: Materials (Basel). 2024 Feb 11;17(4):854. doi: 10.3390/ma17040854 (PMC10890416; doi:10.3390/ma17040854)
Supplement: Supplementary file 1 [file materials-17-00854-s001.zip › materials-2840269-supplementary.pdf]

materials MDPI

Supplementary Materials

Nanocrystals Incorporated MOR Zeolite Composites with Enhanced Upconversion Emission for Cu^{2+} Detection

Peixuan Lin, Song Ye *, Ling Pan, Ruihao Huang, Haoran Zhang, Deping Wang

School of Materials Science and Engineering, Tongji University, Shanghai 201804 China
* Correspondence: yesong@tongji.edu.cn

Table S1. The ion concentrations in the alkaline treatment solution after reaction measured using ICP

Ions Concentration (g/L)	DSi1.0	DSi2.0
Si	3.867	8.367
Al	0.318	0.655

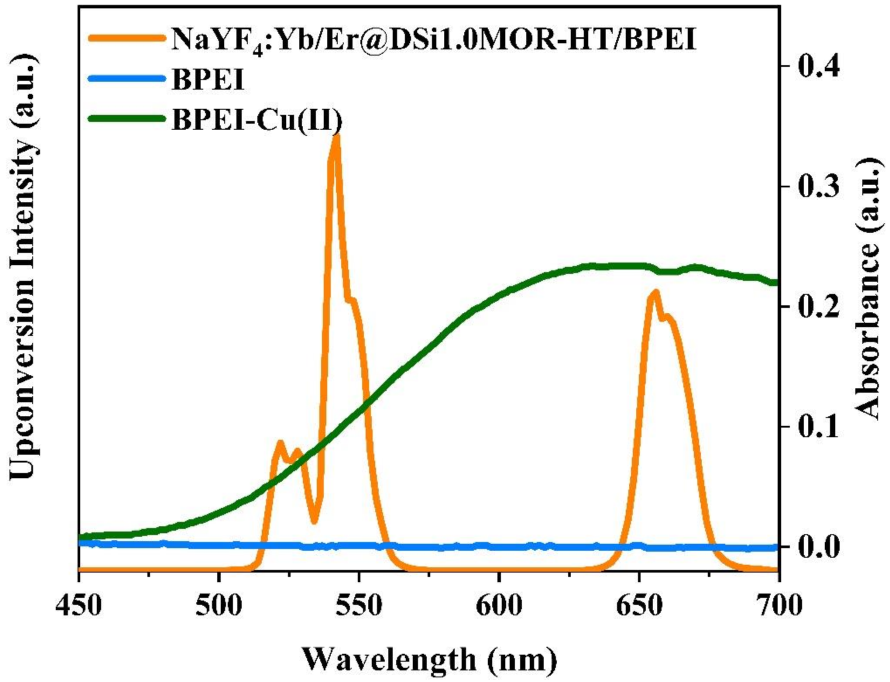

Figure S1. Upconversion spectrum of $NaYF_4$:Yb/Er@DSi1.0MOR-HT composite interacting with BPEI and absorption spectrum of BPEI and BPEI-Cu(II).

Table S2. The limit of detection (LOD) of Cu^{2+}.

Materials used	LODs	Ref.
4-aminoantipyrine-linked bis-1,2,3-triazoles	2.48 mM<br>3.73 mM	[46]
pyridyl-isoindoline-1-one skeleton	2.82 μM	[47]
cyanine-quinone fluorophore	91 μM	[48]

benzotriazole derivative system	8.83 μM<br>8.62 μM<br>2.01 μM	[49]
chemosensor N-(2-hydroxy-3-methoxybenzylidene)-2-(benz-amido) benzohydrazide	1.89 μM	[50]
A morpholine substituted methyl 3-hydroxy-2-naphthoate	2.20 μM	[51]
$NaYF_4$:Yb/Er-RB-hydrazide	1 μM	[52]
$NaYF_4$:Yb/Er @DSiMOR/BPEI	1.507 μM	This work
